# Co-breeding Association of *Aedes albopictus* (Skuse) and *Aedes aegypti* (Linnaeus) (Diptera: Culicidae) in Relation to Location and Container Size

**DOI:** 10.21315/tlsr2018.29.1.14

**Published:** 2018-03-02

**Authors:** Nur Aida Hashim, Abu Hassan Ahmad, Anita Talib, Farida Athaillah, Kumara Thevan Krishnan

**Affiliations:** 1 School of Food Science and Technology, Universiti Malaysia Terengganu, 21030 Kuala Nerus, Terengganu, Malaysia; 2 School of Biological Sciences, Universiti Sains Malaysia, 11800 USM Pulau Pinang, Malaysia; 3 School of Distance Education, Universiti Sains Malaysia, 11800 USM Pulau Pinang, Malaysia; 4 Faculty of Veterinary Medicine, Syiah Kuala University, Banda Aceh, Indonesia; 5 Faculty of Agro Based Industry, Universiti Malaysia Kelantan, Jeli Campus, 17600 Jeli, Kelantan, Malaysia

**Keywords:** *Aedes albopictus*, *Aedes aeypti*, Co-Breeding Association, Shared Breeding Habitat

## Abstract

The occurrence of major outbreaks of dengue, and other vector borne diseases such as chikungunya and zika in tropical and subtropical regions has rendered control of the diseases a top-priority for many affected countries including Malaysia. Control of the mosquito vectors *Aedes aegypti* and *Aedes albopictus* through the reduction of breeding sites and the application of insecticides to kill immature forms and adults are the main control efforts to combat these diseases. The present study describes the association between *Ae. albopictus* and *Ae. aegypti* in shared breeding sites. This study is important given that any measure taken against one species may affect the other. A yearlong larval survey was conducted in four dengue endemic areas of Penang Island. Sorenson’s coefficient index indicated that no association between number of the immatures of the two species regardless of container size and study location. Therefore, the mean number *Ae. albopictus* immature was not decreased in the presence of *Ae. aegypti* in shared breeding container. However *Ae. aegypti* appeared to prefer breeding in habitats not occupied by *Ae. albopictus*, the two species sharing breeding sites only where available containers were limited. In control efforts, eliminating the preferred breeding containers for one species might not affect or reduce the population of the other species.

## Abstract (ru)

Berlakunya wabak denggi, dan lain-lain penyakit bawaan vektor seperti chikungunya dan Zika di kawasan tropika dan subtropika telah menjadikan kawalan penyakit-penyakit tersebut sebagai keutamaan di negara-negara yang terjejas termasuk Malaysia. Kawalan nyamuk vektor *Aedes aegypti* dan *Aedes albopictus* melalui pengurangan tempat pembiakan dan penggunaan racun serangga untuk membunuh jentik-jentik dan nyamuk dewasa adalah usaha utama kawalan bagi memerangi penyakit-penyakit ini. Kajian ini menerangkan tentang hubungan di antara *Ae. albopictus* dan *Ae. aegypti* di dalam tempat pembiakan yang dikongsi bersama. Kajian ini penting memandangkan apa jua langkah kawalan yang diambil terhadap satu spesies boleh menjejaskan spesies yang lain. Tinjauan jentik-jentik selama setahun telah dijalankan di empat kawasan endemik denggi di Pulau Pinang. Indeks pekali Sorenson menunjukkan bahawa tiada perkaitan di antara jumlah jentik-jentik bagi kedua-dua spesies tanpa mengira saiz bekas dan lokasi kajian. Oleh itu, min bilangan jentik-jentik *Ae. albopictus* tidak menurun dengan kehadiran *Ae. aegypti* di dalam bekas pembiakan yang sama. Walau bagaimanapun *Ae. aegypti* lebih gemar membiak di habitat yang tidak diduduki oleh *Ae. albopictus*, kedua-dua spesies hanya berkongsi tempat pembiakan apabila bilangan bekas yang ada adalah terhad. Dalam usaha kawalan, menghapuskan bekas pembiakan yang digemari oleh satu spesies mungkin tidak memberi kesan atau mengurangkan populasi spesies lain.

## INTRODUCTION

A species would not be able to survive on its own but lives together with other organisms to form a community in the same habitat. Co-existence of multiple species of mosquitoes in a habitat at a given time indicates positive interaction among them (Pemola Devi & Jauhari 2007). Interspecific associations among mosquitoes are often related to physicochemical and biological composition of mosquito breeding waters (Reisen *et al.* 1981; Almiron & Brewer 1996; Rajnikant *et al.* 1998). Often, interspecific association shows similarity of habitat requirements and interactions between species (Cole 1949). However, past research on the breeding of different species under field conditions have been based mainly on the frequency of co-occurrence of the immature stages without rigorous statistical analysis to validate the strength of association or repulsion between them (Cole 1949; Bhat 1975a; Bhat 1975b; Malhotra et al. 1987; Bhat *et al.* 1990).

*Aedes albopictus*, is believed to have originated from the tropical forests of Southeast Asia (Smith 1956). Meanwhile, *Ae. aegypti* originated from Africa (Mousson *et al.* 2005). In Malaysia, the first occurrence of *Ae. aegypti* was recorded by Leicester in 1908 and Stanten in 1914 (Lee & Cheong 1987). Population of *Aedes albopictus* and *Ae. aegypti* are common in urban and rural areas of Malaysia (Nazni *et al.* 2009; Saifur *et al.* 2013; Basari *et al.* 2016). Both are sympatric species, and coexist in similar habitat (Klowden 1993; Gilotra *et al.* 1967; Sprenger & Wuithirsnysgool 1986; O’ Meara *et al.* 1993; Chen *et al.* 2006a). Whenever, two species try to coexist in same ecological niches, species replacement or displacement tend to occur. Hawley (1988) reported that species replacement occurred in particular in the North of America, where *Ae. aegypti* abundance had been reduced as a result of competition with *Ae. albopictus*. Whereas, Juliano (1998) reported displacement of *Ae. aegypti* by *Ae. albopictus* which might due to larval competition on available food resource. Others reported replacement of *Ae. albopictus* by *Ae. aegypti* in the peripheral areas of towns of India (Kalra *et al.* 1997).

On the contrary, others reported *Ae. aegypti* has completely replaced the indigenous *Ae. albopictus* in urban areas (Pant *et al.* 1973; Service 1992). In Bangkok, Thailand and Calcutta, India, *Ae. albopictus* had decreased in population, while *Ae. aegypti* has become more pronounced (Rudnick & Hammon 1960; Gilotra *et al.* 1967). In addition, experiments conducted in controlled environment support the proposition that *Ae. aegypti* can out-compete and displace *Ae. albopictus* (Moore & Fisher 1969; Sucharit *et al.* 1978; Black *et al.* 1989). Lambrechts *et al.* (2010) hypotesize, Ae. aegypti is gradually replacing Ae. albopictus as the dominant day-biting mosquito in Asian cities because it is better adapted to the urban environment.

In Penang Island, Malaysia, during the year 1970’s, *Ae. aegypti* was not documented beyond the city limit of Georgetown to the rest of the island (Yap 1975). However, recent studies, shows that the species is observed in rural and urban residential areas of the island (Saifur *et al.* 2013). Compared with field study done in Northern Queensland, Australia, it was noted that *Ae. notoscriptus* container distribution was affected by the presence or absence of *Ae. aegypti* although they found no association in the relative abundance of both species (Tun Lin *et al.* 1999). In summary, researchers noted *Ae. aegypti* and *Ae. albopictus* do mix-breed in large-sized water containers regardless whether it is an indoor or outdoor environments (Hwang & Hsu 1994; Chen *et al.* 2006a).

Therefore, for an effective mosquito control regime, the relationship between habitats, environmental factors and occurrence of immature mosquitoes must be well understood. The association between species of mosquitoes can provide clues to better understanding of their biology and roles in the transmission of the vector borne viruses such as dengue. Therefore, it is important to determine the strength of association of these two species, in respect of positive association (overlapping), negative association (repulsion) or zero association (the species is independent). This study sought to determine if there was co-breeding association between *Ae. aegypti* and *Ae. albopictus* in shared breeding containers in the Southwest district of Penang, Malaysia.

## MATERIALS AND METHODS

### Study Sites

**Figure 1:**
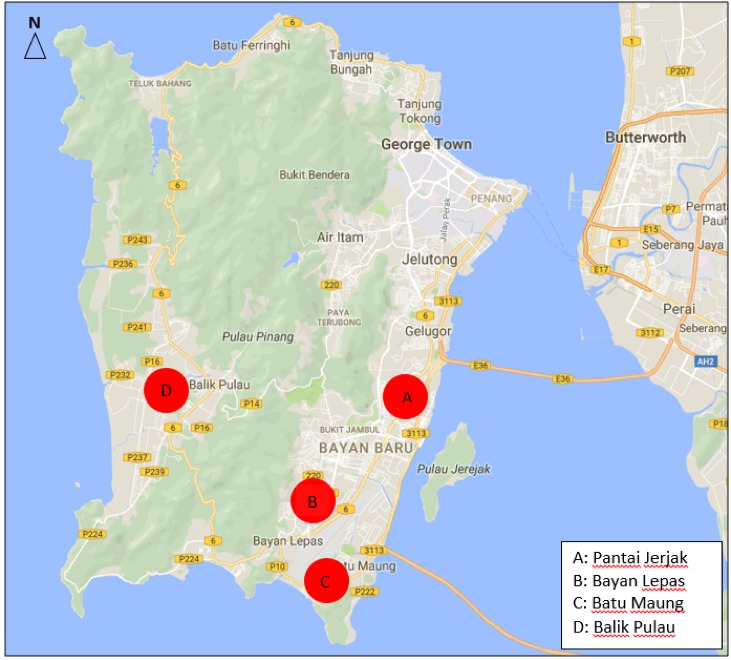
Location of sampling sites, Pantai Jerjak, Bayan Lepas, Batu Maung and Balik Pulau, Penang. Source: https://www.google.com.my/maps

Four areas within Southwest district of Penang Island were selected for this study (Fig. 1). The areas were: Pantai Jerjak (urban residential area) located at 5.337681° N, 100.302187° E (12 m.a.s.l.), Bayan Lepas (urban residential/industrial area) located at 5.298113° N, 100.262276° E (14 m.a.s.l.), Batu Maung (suburban residential area) located at 5.274604° N, 100.267525° E (8 m.a.s.l.) and Balik Pulau (rural area) located at 5.325033° N, 100.212108° E (18 m.a.s.l). Climatological data for Penang Island including rainfall, mean relative humidity and mean temperature were obtained from the Malaysian Meteorological Station located at Penang International Airport.

### Larval Survey

Larvae collection was done for 12 months (January 2009 to December 2009). The sampling were performed on monthly basis in each study area stated above by three two-person collection teams (between 0900 h and 1500 h). A total of 720 houses in each study area were inspected for mosquito breeding sites. During sampling, an inspection of the domestic and peri-domestic area of each house in study areas for water holding containers was performed. The containers were categorised into three sizes: small (capacity < 1 litre), medium (1 litre < capacity < 15 litres) and large (capacity > 15 litres). Due to the different sizes of the containers, sampling methods for the three container categories also differed. For small containers, all the contents of the containers were poured into zip-lock plastic bags, while for medium and large containers only the *Aedes* immatures (pupae and larvae) were collected using pipette or sieves and placed into zip lock plastic bags. All the water samples (in plastic bags) were labelled with house description and container name so that samples could be linked to the exact container and household of origin. The containers with mosquitoe’s larvae were also classified into three categories:

- Single container – with either *Ae. albopictus* or *Ae. aegypti*
- Shared container – *Ae. albopictus* and *Ae. aegypti* together
- Other container – other mosquito species, *Aedes* absent

The samples were transported to the laboratory at the School of Biological Sciences, Universiti Sains Malaysia on the same day they were collected for further processing. For the purpose of identification, pupae were reared and identified when they developed into adults. The 1st and 2nd instar larvae were allowed to moult to the 3rd and 4th instar to facilitate identification; 3rd and 4th instar were identified to the species level using taxonomic keys provided by Rueda (2004) under a dissecting microscope (Olympus CX41, Olympus, Tokyo, Japan).

### Data Analyses

The Chi-square test was used to analyse differences in container abundance, immature abundance, immature species and container sizes using the SPSS version 21.0.

### Coefficient of Interspecific Association

The method of Fager (1957) as detailed by Southwood (1978) was used to explain the independence or association of the two species. An association between *Ae. albopictus* and *Ae. aegypti* would be determined from the proportion of positive containers containing *Ae. albopictus* in the presence or absence of *Ae. aegypti* in the same containers. If there is no association, the same proportions of *Ae. aegypti* should be observed irrespective of whether *Ae. albopictus* was present or not (Tun Lin *et al.* 1999).

To calculate the coefficient of association, 2 × 2 contingency tables were drawn up where a, b, c and d were the number of occurrences of the two species in water containers as shown in the table below, where species A is the more abundant species.

**Table:** 

Species A				

		present	absent	Totals
	present	a	b	a + b
Species B	absent	c	d	c + d
	Total	a + c	b + d	n = a + b + c + d

Where, a = the presence of both species (A and B) in shared containers, b = the presence of species A but species B absent, c = the presence of species B but species A absent, d = samples of other mosquito species but species A and B absent.

In this case, counts for the more abundant species, *Ae. albopictus* (species A) occupy cells a and c, whereas counts for *Ae. aegypti* (species B) occupy cells c and d. Accordingly, (a+b) < (a+c). Cell d (neither *Ae. albopictus* nor *Ae. aegypti* present) was calculated on the basis of the positive containers only and not on the total number of wet negative containers. The table was constructed in Microsoft Excel workbook (version 2010) and statistically significant differences were calculated by the Chi square test as corrected by Pielou (1977):

$${\text{X}}^{2}=\frac{{\left[|\text{ad}-\text{bc}|-\text{N}/2\right]}^{2}\;\text{N}}{\text{mnrs}}$$

Where, m = (a+b), n = (c + d), r = (a + c), s = (b + d) and N = m + n + r +s

### Index of Association (I)

The proportion of individuals occurring together was calculated using Sorensen’s Coefficient Index (1948) as modified by Southwood (1978). The formula was as follows:

I=2 [J/(A+B)-0.5]

where

J = the number of *Ae. albopictus* and *Ae. aegypti* immatures where the two species shared positive containers,
A = the number of *Ae. albopictus* immature found in all positive containers
B = the number of *Ae. aegypti* found in all positive containers.

An Index value of +1 indicates complete association while −1 indicates no association.

### Dominance Index (D)

Species dominance, D, using May’s (1975) index was calculated for each study site and container size:

$$\text{D}={\text{Y}}_{\text{max}}/{\text{Y}}_{\text{t}}$$

Where Y_max_ = the number of larvae of the most common species (*Ae. albopictus*) in the each study site or each container size, Y_t_ = the total numbers of larvae of all species in the habitat.

## RESULTS

During the larval survey, the monthly mean temperature and mean relative humidity in Penang Island ranged between 26.0°C to 28.0°C and 59% to 89% respectively. Overall, Penang received a total rainfall of 2407.6 mm.

Table 1 shows co-breeding association between *Ae. albopictus* and *Ae. aegypti* in the four study areas. The distribution of *Ae. albopictus* positive containers combined for the four sampled areas was significantly different χ^2^_(1,1567)_ = 558.52, *p* < 0.05 in the presence or absence of *Ae. aegypti*. There was also significant co-breeding interaction between *Ae. albopictus* and *Ae. aegypti* container distribution at each area [Pantai Jerjak, χ^2^_(1,296)_ = 147.97, *p* < 0.05; Bayan Lepas, χ^2^_(1,387)_ = 151.29, *p* < 0.05; Batu Maung, χ^2^_(1,388)_ = 122.48, *p* < 0.05; Balik Pulau, χ^2^_(1,496)_ = 52.29, *p* < 0.05].

When the mosquitoes population were compared by container sizes, it showed significant co-breeding interaction between *Ae. albopictus* and *Ae. aegypti* distribution in containers of different sizes [ Small, χ^2^_(1,818)_ = 94.51, *p* < 0.05; Medium, χ^2^_(1,521)_ = 211.55, *p* < 0.05; Large: χ^2^_(1,228)_ = 107.20, *p* < 0.05] (Table 2).

However, when comparison were made, in terms of the abundance of immatures, there was no co-breeding association between *Ae. albopictus* and *Ae. aegypti* in each study area (Table 3). Similarly, the analysis between *Ae. albopictus* and *Ae. aegypti* immature relative abundance in three different sizes of containers indicated no co-breeding association between both species in each container size (Table 4).

Tables 5 and 6 shows the species dominance index calculated for each study area and container size, respectively. The results showed that *Ae. albopictus* was the dominant species (> 90%) for all study areas. Thus, *Ae. albopictus* is the dominant *Aedes* species in the Southwest district of Penang Island, regardless whether it is urban, suburban or rural area.

## DISCUSSION

According to Hurlbert (1969), the analysis of presence-absence data is preferable to that of the relative number of immature stages for measuring the degree of association between two species. However, Southwood (1978) suggested to employ both methods. Positive association means two species interact in such a way as to favour mutual presence. Negative association is to be anticipated when one species exclude the other from the habitat.

In the present study, *Ae. albopictus* and *Ae. aegypti* were found in single and shared containers regardless of the geographical characteristics (urban, suburban) and container size (small, medium, large). Negative value for (ad–bc) (Table 2) indicated that there was a negative association between the two species which indicated that *Ae. aegypti* preferred to fill in habitats which were not occupied by *Ae. albopictus*. It is possible that after entering houses to blood-feed, *Ae. albopictus* found indoor containers which had been occupied by *Ae. aegypti* when water holding containers outdoor dried out during the dry season. Past research observed *Ae. albopictus* do oviposit indoors in human dwellings (Sulaiman *et al.* 1991; Chen *et al.* 2006b; Lian *et al.* 2006; Wan-Norafikah *et al.* 2010; Dieng *et al.* 2010) and the most anthropophilic mosquito in Malaysia (Parker *et al.* 1983).

Negative co-breeding association between the two species in all container sizes confirmed that *Ae. albopictus* would fill out niches unoccupied by *Ae. aegypti*. The latter prefers to breed in both indoor and outdoor containers where vegetation in the areas are less. Previous studies showed *Ae. aegypti* to be the dominant indoor species (Surendran *et al.* 2007; Singh *et al.* 2008; Wan-Norafikah *et al.* 2010). According to Gilotra *et al.* (1967), *Ae. aegypti* is the superior competitor in domestic premises, whereas *Ae. albopictus* has the advantage in outdoor or silvatic surroundings.

The Sorenson’s Coefficient Index showed there was no significant association between individual immature for the two species and no association in relative abundance between individual species. Similar results were obtained in all the study areas and each container size. The index value for urban and suburban was similar suggesting dominance of *Ae. albopictus* in small and medium size containers. The mean immature densities of *Ae. albopictus* were not depressed significantly in the presence of *Ae. aegypti*. *Aedes albopictus* continued to be the dominant *Aedes* species in the Southwest district of Penang Island despite the spread of *Ae. aegypti* out of the city limit. Similarly, Tun-Lin *et al.* (1999) found that there was a significant co-breeding association in the distribution of positive containers for *Ae. notocriptus* depending on the presence and the absence of *Ae. aegypti* in Australia. They also found that there was little or no association between the two species in their relative abundance of immatures in shared containers.

Being the dominant *Aedes* species in the Southwest district of Penang Island, *Ae. albopictus* might play an important role in the transmission of dengue and chikungunya viruses. According to Lounibos (2002), though *Ae. aegypti* is the main dengue vector, *Ae. albopictus* is also a competent vector and may be locally important. Interspecies competition between larvae change *Ae. albopictus* behaviour. In shared breeding habitat, the larvae adapted by swimming faster, increased their movement and feeding rate. Breeding containers with high larvae density tend to have limited space and resource. Therefore, *Ae. albopictus* larvae that have less food during development will emerge as an adult smaller in size which was reported to affect its fitness, reproductive rate and capacity as a vector (Blaustein *et al.* 2005; Preisser *et al.* 2005; Bara *et al.* 2015).

For *Ae. albopictus*, competition increases the probability of obtaining arboviruses (Alto *et al.* 2005; Alto *et al.* 2008) and competition among larvae may affect the probability of vector-borne virus transmission (Alto *et al.* 2008). Furthermore, effects of competitive interactions among larval stages may be carried over to the adult stage and affect vector competence, which describes the ability to become infected and subsequently to transmit a pathogen after imbibing an infectious blood meal (Hardy 1988).

When comparing larval competition in co-exist populations, *Ae. aegypti* stand a better chance as it requires shorter developmental time than *Ae. albopictus* (Chan *et al.* 1971). However, Phon (2007) indicated the competitive advantage of *Ae. albopictus* in situations of limited resources could be the reason for the dominancy of this mosquito in Penang Island. Barrera (1996) noted the presence of rapidly decaying detritus (e.g., animal detritus) tends to yield competitive equality or advantage for *Ae. aegypti*, whereas refractory plant detritus (deciduous or coniferous leaves) tends to yield competitive advantage for *Ae. albopictus.* He also emphasized the interspecific differences in starvation resistance of larvae of these species also depended on type of food resource. *Aedes albopictus* and *Ae. aegypti* were found to withstand starvation when reared on oak leaves and liver powder, respectively, suggesting a physiological basis for the detritus-type-dependence having an impact on co-breeding competition of these two species.

In Australia, Tun-Lin *et al.* (1999) proposed the association between *Ae. aegypti* and *Ae. notoscriptus* could be due to competitive displacement of immature stages, different adult ovipositional stimuli or pheromonal repellents. However, competitive displacement of *Ae. albopictus* by *Ae. aegypti* in Penang Island is unlikely to happen. Shared breeding between *Ae. albopictus* and *Ae. aegypti* encountered in the present study was very low. The present study demonstrated that there was negative co-breeding association between the two species in their container distribution (number of container) and no association existed between the number of immatures of both species. Therefore, statistically, the interaction was significant only in the number of containers occupied by both species but there was no interspecies association from the perspective of individual mosquitoes.

The spread of *Ae. aegypti* in Penang Island could be due to several factors such as the rapid and extensive urbanisation of the city, the difference in fecundity between *Ae. aegypti* and *Ae. albopictus* and the difference in the duration of the life cycle of the two species. Favourable environment for the highly domesticated *Ae. aegypti* has been created with rapid and extensive urbanisation, and this condition leading to the rapid spread and increase in numbers of the species. However, *Ae. albopictus* probably has never been displaced by *Ae. aegypti* from the urban areas since the current trend of urban development is towards a ‘garden city’ where habitats would still be available for *Ae. albopictus* (Chan *et al.* 1971).

## CONCLUSION

Negative interspecific association was observed between *Ae. albopictus* and *Ae. aegypti* in breeding containers in four survey areas of Southwest district on Penang Island suggesting *Ae. aegypti* distribution is restricted by *Ae. albopictus*. In addition, though *Ae. albopictus* and *Ae. aegypti* share the same breeding habitat, both prefer different environments (indoor or outdoor). The two species would avoid breeding in the same containers. Therefore, as the two species have different preferences in the selection of breeding environment, mosquito control should be emphasised in both inside and outside areas.

## Figures and Tables

**Table 1:** Distribution of *Ae. albopictus* and *Ae. aegypti* from positive containers found in Penang Island.

Survey	Species	*Ae. aegypti* (A)					

			Presence	Absence	Total	ad – bc (−ve/+ve)	χ2
All sites (4 study areas combined)	*Ae. albopictus* (B)	Presence	57 a	106 b	163	−ve	χ^2^ = 558.52^*^
		Absence	1343 c	61 d	1404		
		Total	1400	167	1567		
Pantai Jerjak	*Ae. albopictus*	Presence	15	37	52	−ve	χ^2^ = 147.97^*^
		Absence	236	8	244		
		Total	251	45	296		
Bayan Lepas	*Ae. albopictus*	Presence	19	33	52	−ve	χ^2^ = 151.29^*^
		Absence	323	12	335		
		Total	342	45	387		
Batu Maung	*Ae. albopictus*	Presence	21	30	51	−ve	χ^2^ = 122.48^*^
		Absence	322	15	337		
		Total	343	45	388		
Balik Pulau	*Ae. albopictus*	Presence	2	6	8	−ve	χ^2^ = 52.29^*^
		Absence	462	26	488		
		Total	464	32	496		

Note: ^*^significant, *p* < 0.05, Where, **a** = the presence of both species (A and B) in shared containers, **b** = the presence of species A but species B absent, **c** = the presence of species B but species A absent, **d** = samples of other *Aedes* species but species A and B absent, **bc** = single containers for both species, **ad** = both species in shared containers and negative containers, positive association when **ad-bc** = +ve, negative association/repulsion when **ad-bc** = −ve

**Table 2:** Distribution of *Ae. albopictus* and *Ae. aegypti* from positive containers of different sizes.

Survey	Species	*Ae. aegypti* (A)					

			Presence	Absence	Total	ad – bc (−ve/+ve)	χ2
All sites (4 study areas combined)	*Ae. albopictus* (B)	Presence	57 a	106 b	163	−ve	χ^2^ = 558.25 ^*^
		Absence	1343 c	61 d	1404		
		Total	1400	167	1567		
Small	*Ae. albopictus*	Presence	21	20	41	−ve	χ^2^ = 94.51 ^*^
		Absence	733	44	777		
		Total	754	64	818		
Medium	*Ae. albopictus*	Presence	18	33	51	−ve	χ^2^ = 211.55 ^*^
		Absence	457	13	470		
		Total	475	46	521		
Large	*Ae. albopictus*	Presence	18	53	71	−ve	χ^2^ = 107.20 ^*^
		Absence	153	4	157		
		Total	171	57	228		

Note; ^*^significant, *p* < 0.05, Where, **a** = the presence of both species (A and B) in shared containers, **b** = the presence of species A but species B absent, **c** = the presence of species B but species A absent, **d** = samples of other *Aedes* species but species A and B absent, **bc** = single containers for both species, **ad** = both species in shared containers and negative containers, positive association when **ad–bc** = +ve, negative association/repulsion when **ad–bc** = −ve.

**Table 3:** Sorenson coefficient of interspecific association between *Ae. albopictus* and *Ae. aegypti* immature in four survey areas on Penang Island.

Survey Areas	J	A + B	I
Pantai Jerjak	1735	14828	−0.77
Bayan Lepas	1257	21306	−0.88
Batu Maung	2533	16045	−0.68
Balik Pulau	61	24196	−0.99
Combined (all sites)	5586	76375	−0.85

Note: Significant association when I = +1, No association when I = −1

**Table 4:** Sorenson coefficient of interspecific association between *Ae. albopictus* and *Ae. aegypti* immatures in containers of three different sizes.

Size	J	A+B	I
Small	1508	22499	−0.87
Medium	2599	25877	−0.8
Large	1479	27999	−0.89
Combined	5586	76375	−0.85

Note: Significant association when I = +1, No association when I = −1

**Table 5:** Species dominance index in the four survey areas on Penang Island.

Survey Areas	Y_max_	Y^t^	D
Pantai Jerjak	13275	14828	0.90
Bayan Lepas	20250	21306	0.95
Batu Maung	14468	16045	0.90
Balik Pulau	23880	24196	0.99
Combined	71873	76375	0.94

Notes: Y_max_ = the number of immatures of the most common species (*Ae. albopictus*) in each survey areas, Y_t_ = the total number of immatures of all species in the areas.

**Table 6:** Species dominance index in containers of three different sizes.

Size	Y_max_	Y_t_	D
Small	21769	22499	0.97
Medium	24608	25877	0.95
Large	25496	27999	0.91
Combined	71873	76375	0.94

Where Y_max_ = the number of immatures of the most common species (*Ae. albopictus*) in each survey areas, Y_t_ = the total number of immatures of all species in the areas.
